# Additive effects of *p*CO_2_ and temperature on respiration rates of the Antarctic pteropod *Limacina helicina antarctica*

**DOI:** 10.1093/conphys/cox064

**Published:** 2017-11-29

**Authors:** Umihiko Hoshijima, Juliet M Wong, Gretchen E Hofmann

**Affiliations:** 1 Department of Ecology, Evolution, and Marine Biology, University of California Santa Barbara, Santa Barbara, CA 93106-9620, USA

**Keywords:** *Limacina helicina*, metabolic rate, ocean acidification, pH, pteropods, temperature

## Abstract

The Antarctic pteropod, *Limacina helicina antarctica*, is a dominant member of the zooplankton in the Ross Sea and supports the vast diversity of marine megafauna that designates this region as an internationally protected area. Here, we observed the response of respiration rate to abiotic stressors associated with global change—environmentally relevant temperature treatments (−0.8°C, 4°C) and pH treatments reflecting current-day and future modeled extremes (8.2, 7.95 and 7.7 pH at −0.8°C; 8.11, 7.95 and 7.7 pH at 4°C). Sampling repeatedly over a 14-day period in laboratory experiments and using microplate respirometry techniques, we found that the metabolic rate of juvenile pteropods increased in response to low-pH exposure (pH 7.7) at −0.8°C, a near-ambient temperature. Similarly, metabolic rate increased when pteropods were exposed simultaneously to multiple stressors: lowered pH conditions (pH 7.7) and a high temperature (4°C). Overall, the results showed that *p*CO_2_ and temperature interact additively to affect metabolic rates in pteropods. Furthermore, we found that *L. h. antarctica* can tolerate acute exposure to temperatures far beyond its maximal habitat temperature. Overall, *L. h. antarctica* appears to be susceptible to pH and temperature stress, two abiotic stressors which are expected to be especially deleterious for ectothermic marine metazoans in polar seas.

## Teaser

Swimming marine snails in the Antarctic are a key component of the pelagic community. They exhibit higher respiration rates under future ocean scenarios of low pH when compared to current-day conditions. They also exhibit higher respiration rates under future low-pH conditions when paired with a high temperature stress.

## Introduction

Organismal responses to singular abiotic stressors have long been utilized to investigate the role of physiological tolerance in shaping ecosystems and communities (Somero, 2002). However, assessing physiological conditions in response to multiple environmental stressors has recently become recognized as a critical step towards predicting how populations will persist in the future in response to anthropogenic environmental change (Todgham and Stillman, 2013; Boyd *et al.*, 2015; Gunderson *et al.*, 2016). As climate change will involve complex physical factors changing together (Gunderson *et al.*, 2016), experiments investigating environmentally relevant multistressor exposures are quickly becoming a critical component of global change biology.

In line with the process of global climate change (GCC), there is an emergent need to find species which can be used to assess ecosystem health. These organisms, often referred to as indicator species, can allow for a better understanding of global ocean conditions, especially in remote locations, as long as the species is well-studied in the context of these global stressors (Landres *et al.*, 1988). Certain animals may also act as valuable sentinel organisms, helping to indicate the first signs of GCC impacts on ecosystems (Bednaršek *et al.*, 2014; Johnson and Hofmann, 2017; Manno *et al.*, 2017). For instance, thecosome pteropods possess a very fragile shell composed primarily of aragonite (Orr *et al.*, 2005; Comeau *et al.*, 2010), a more soluble form of CaCO_3_ that is particularly vulnerable to ocean acidification (OA) (Mucci, 1983; Comeau *et al.*, 2010). Previous studies have shown evidence of shell dissolution and malformations under future *p*CO_2_ levels (Orr *et al.*, 2005; Lischka *et al.*, 2010), which may adversely impact the shell’s protective capacity against predators or infection, or the shell’s ballast ability, which aids in vertical migration (Comeau *et al.*, 2010). Through this lens, the overarching goal of this study was to examine the response of an important member of the zooplankton in the Southern Ocean, the pteropod *Limacina helicina antarctica*, to temperature changes and simulated OA.

Although it is well understood that GCC and OA are drastically increasing global sea surface temperatures and reducing the mean surface ocean pH levels at an accelerated rate (0.6–2.0°C increase and a 0.3–0.32 pH units decrease by the year 2100) (IPCC, 2013), changes in ocean conditions are predicted to be particularly dramatic in polar seas (Steinacher *et al.*, 2009) with marine communities in the Arctic and Southern Ocean expected to experience significant changes in physiochemical conditions in this century (Hauri *et al.*, 2016). In particular, the Southern Ocean has already warmed at a rate almost double that of the global trend in the upper 1000 m of ocean waters (0.17°C in the Southern Ocean between the 1950s and the 1980s) (Levitus *et al.*, 2000; Gille, 2002; Fyfe, 2006). Additionally, the surface waters of the Southern Ocean are inherently susceptible to OA due to the high solubility of gases in cold waters, with surface ocean undersaturation of aragonite (Ω_arag_ < 1) expected to develop by the year 2030 (McNeil and Matear, 2008; Hauri *et al.*, 2016). Considering these observed trends, understanding how Antarctic marine organisms respond to the combined impacts of warming and acidification is of paramount importance for predicting how these factors will impact the unique ecosystem that persists in the Southern Ocean (Boyd *et al.*, 2016).

In the context of future ocean change, organisms that inhabit the Southern Ocean may be limited in their ability to adapt or migrate. Polar ectotherms often possess slow growth rates and long generation times, which generally result in a lower capacity for adaptation (Peck, 2005; Pörtner *et al.*, 2007). Furthermore, unlike some temperate and tropical species that have been observed to migrate poleward with increasing oceanic temperatures (Sorte *et al.*, 2010; Sunday *et al.*, 2012; Poloczanska *et al.*, 2013), polar species are already restricted to high latitudes and will more likely undergo range contractions with continuing GCC (Whiteley, 2011). In addition to being stenothermal, possessing a narrow range of thermal tolerance from adapting under very stable conditions for millions of years (Peck, 2005; Sorte *et al.*, 2010; Somero, 2012), these organisms may potentially be living at or near their thermal tolerance maxima (Whiteley, 2011).

In contrast, seasonal pH variation in the Ross Sea (Kapsenberg *et al.*, 2015) indicates that Antarctic species may have more physiological tolerance and potential for adaptation to OA stress than expected. Indeed, larvae of the sea urchin, *Sterechinus neumayeri*, were found to be remarkably tolerant to OA and warming (Kapsenberg and Hofmann, 2014), although other studies have shown sensitivity of calcification processes in early stages (Byrne *et al.*, 2013). Stress responses, however, can vary greatly by species (Byrne and Przeslawski, 2013; Breitburg *et al.*, 2015; Przeslawski *et al.*, 2015; Stillman and Armstrong, 2015). Additionally, although variability in pH has been characterized across a variety of spatiotemporal scales (Hofmann *et al.*, 2011), the high seasonality of pH in Antarctic surface waters indicates the potential for spawning phenology of polar organisms to be a critical factor in assessing the exposure of early life stages to near-future OA (Kapsenberg *et al.*, 2015).

In the Southern Ocean, *L*. *h. antarctica* is a dominant member of the zooplankton (Hunt *et al.*, 2008). Thecosome pteropods are a key study organism in polar regions, holding an essential role in food web dynamics and energy transfer (Willette *et al.*, 2001; Hunt *et al.*, 2008; Bednaršek *et al.*, 2012) by acting as major consumers of phytoplankton (Perissinotto, 1991; Bernard and Froneman, 2009) while serving as prey for a variety of organisms, including gymnosome pteropods, euphausids, salps, and a multitude of pelagic and demersal fish species (Lancraft *et al.*, 1991; Hunt *et al.*, 2008). As such, *L. h. antarctica* is a key player in the simple, yet fragile Antarctic food web and directly impacts the food sources of higher trophic levels, including predatory birds and mammals, such as penguins, whales and seals (Davis *et al.*, 1999; Seibel and Dierssen, 2003; McClintock *et al.*, 2008). Thecosome pteropods also play a sizeable role in geochemical cycling of both organic and inorganic carbon, producing fecal pellets and calcium carbonate (CaCO_3_) shells that sink to deeper waters (Berner and Honjo, 1981; Fabry, 1990; Gilmer and Harbison, 1991; Collier *et al.*, 2000; Tsurumi *et al.*, 2005; Manno *et al.*, 2009). Manno *et al.* (2009) estimated that *L. h. antarctica* could contribute as much as 72% of the total organic carbon export in the Ross Sea, and Honjo (2004) found that south of the Polar Front, pteropods were the main contributor to an inorganic CaCO_3_ flux of 110 mmol C m^−2^ yr^−1^. Overall, *L. h. antarctica* holds a key role in Southern Ocean ecosystems, but unfortunately, changing ocean temperatures and pH levels may adversely impact this species. Due to this considerable abundance and ecological importance of *L. h. antarctica* in the Southern Ocean, as well as its potential vulnerabilities to elevated temperatures and *p*CO_2_ levels, it is critical to investigate how this species copes with multiple abiotic stressors and consequently, the capacity of these populations to withstand dynamic environmental change.

To complicate matters, the interaction effect between *p*CO_2_ and temperature stress can vary drastically, and can have key physiological consequences (Todgham and Stillman, 2013). An additive interactive effect may occur if each individual stressor does not interact with one another, or if one or more of the stressors has no significant effect—as *p*CO_2_ and temperature have been observed in certain mollusk species (Talmage and Gobler, 2011). However, other organisms, such as sea urchin larvae, have been shown to respond to these same two stressors antagonistically when the negative effects of one stressor are offset by the other (Sheppard Brennand *et al.*, 2010). Lastly, the two stressors may exacerbate one another, resulting in a synergistic interaction effect between stressors as has been observed in the Arctic pteropod (Lischka and Riebesell, 2012). Understanding the interaction of environmentally co-occurring stressors in sentinel species, especially in areas of the world’s oceans potentially most vulnerable to global change, is thus a key research priority in global change biology.

Here, we examined the nature of two key multistressors, *p*CO_2_ and temperature, on the respiration rates of the Antarctic pteropod *L. h. antarctica*. Interrogating metabolic rate as a proxy for organismal energy dynamics is a useful strategy in global change studies and can provide valuable insight into physiological state (Sokolova, 2013). Hypothetically, under OA stress alone, pteropods may increase their metabolism to support shell repair, or to maintain calcification under elevated *p*CO_2_ conditions. In addition to shell effects, elevated *p*CO_2_ levels can also alter physiology by interacting with internal and external body fluids, causing acid-base imbalances and reduced capacity for oxygen transport (Fabry *et al.*, 2008; Melzner *et al.*, 2009; O’Donnell *et al.*, 2010). Metabolic suppression has been identified as an adaptive mechanism by which some organisms cope with hypercapnia, but prolonged metabolic suppression will have consequences on growth and survival (Fabry *et al.*, 2008; Dymowska *et al.*, 2012). In contrast, some species appear to respond to OA by increasing their metabolic rates, although this comes at an energetic cost (Wood *et al.*, 2008; Stumpp *et al.*, 2011). Increased temperatures will also increase metabolic rates due to enzyme kinetics based on the Boltzmann–Arrhenius model (Dell *et al.*, 2011), which may interact with the consequences of a high *p*CO_2_ environment (Gunderson *et al.*, 2016).

In nature, individuals of *L. h. antarctica* may modify their metabolic rate as a result of seasonal and developmental changes. *L. h. antarctica* have a lifespan of 1–3 or more years (Hunt *et al.*, 2008; Bednaršek *et al.*, 2012) in which juveniles overwinter before maturing into adults in the early summer (Gannefors *et al.*, 2005; Hunt *et al.*, 2008). Overwintering juveniles may be especially susceptible to elevated *p*CO_2_ levels and temperatures because, during the wintertime when algal food supplies are limited, polar organisms are known to lower their metabolism as a means of conserving energy (Hirche, 1996; Hagen and Auel, 2001), possibly decreasing their resistance to abiotic stressors (Lischka *et al.*, 2010). Maas *et al.* (2011) demonstrated that *L. h. antarctica* lowered its metabolic rate, measured by a decrease in oxygen consumption, under conditions of reduced food availability. Over the winter, the Arctic pteropod subspecies, *L. helicina helicina*, temporarily stops growing, perhaps lowering its activity and calcification processes while relying on internal lipid stores (Lischka and Riebesell, 2012). Metabolic suppression may decrease an organism’s ability to cope with OA and/or warming, or else metabolism must be increased in order to respond, effectively forcing the use of energy reserves at a time when conserving energy is a necessity (Lischka and Riebesell, 2012). Thus, research on juvenile *L. h. antarctica* provides valuable information on what is potentially its most vulnerable life history stage (Manno *et al.*, 2017).

Here, we examined the response of juvenile Antarctic pteropods, *L. h. antarctica*, to abiotic stressors related to both temperature and pH by measuring their metabolic rate under laboratory treatments reflective of current and future Southern Ocean abiotic conditions. We also describe an experimental setup designed to maintain pteropods under controlled *p*CO_2_ and temperature conditions. Using a microplate respirometry system, oxygen consumption measurements were recorded during 2-week exposures to a combination of three *p*CO_2_ levels and two temperatures chosen to reflect current and predicted future ocean conditions during both winter and summer seasons in this region. Additionally, oxygen consumption rates were measured along a range of elevated temperatures to determine the critical thermal maxima (CT_max_). Due to their reliance on their relatively vulnerable aragonitic shell, our goal was to examine the impacts of differential *p*CO_2_ and temperature conditions on juvenile *L. h. antarctica* by assessing degrees of metabolic change.

## Materials and methods

### Field collection of pteropods

Pteropods were collected ~1 km from shore near McMurdo Station, Antarctica at a research hut site on first-year fast ice (77°50′54″ S, 166°35′55″ E) with a maximum water depth of 170 m. Collections were made using a fixed-frame bongo net (two 50 cm diameter × 150 cm length nets with 333 μm mesh and cod ends with 200 and 333 μm mesh) deployed to a depth of 50 m through a 1.3 m diameter hole drilled in the sea ice. Floats attached to each cod end minimized pinching of the net, while a swiveling double shackle allowed the net to rotate and tilt along with the water currents. The duration of each bongo net deployment was approximately 24 h. Collections were performed throughout the 2015 austral spring and summer, and selected tows supported three experiments described in this study: (i) an experiment run at ambient temperature, (ii) an experiment run at an elevated temperature, 4°C and (iii) an experiment to determine the thermal tolerance of *L. h. antarctica* (referred to as E1, E2 and E3, respectively). E1 pteropods were collected on the 21st and 22nd of October, E2 pteropods on the 11th of November, and E3 pteropods on the 30th of November. Following collection, pteropods were immediately transported to the Crary Lab aquarium facilities at McMurdo Station, where they were held in 500 mL polycarbonate Nalgene containers filled with seawater at ~ −0.8°C for no longer than 1 day until the start of each experiment.

### Experimental design

For CO_2_ exposure experiments (E1 and E2), pteropods were held in a flow-through zooplankton culturing system with controlled levels of *p*CO_2_ while maintaining sufficient flow for long-term experiments. The reservoir mixing system that generated the target experimental *p*CO_2_ levels was constructed following Fangue *et al.* (2010). Briefly, filtered, CO_2_-scrubbed (Sodasorb, Smith’s Medical, St. Paul, MN, USA), dried (W.A. Hammond Drierite Co, Stock no. 23 001, Xenia, OH, USA) air was mixed with pure CO_2_ using SmartTrak™ 100L Series Mass Flow Controllers and MicroTrak™ 101 Series Mass Flow Controllers (Sierra Instruments, Monterey, CA, USA), respectively. These reservoirs were held in a sea table that kept the treated seawater at the desired experimental temperature (~ −0.8°C for E1 and ~ 4°C for E2) to represent the current average temperature and an elevated temperature based on the Representative Concentration Pathway (RCP) 8.5, the scenario that represents the highest greenhouse gas emissions (IPCC, 2013, Moss *et al.*, 2010, Riahi *et al.*, 2011). The *p*CO_2_ treated seawater was then pumped into the zooplankton flow-through culturing system.

Acidification treatments were chosen to represent the range of current monthly means of pH in McMurdo Sound (measured 2 km away from the pteropod collection site), as well as modeled end-of-century pH based on an equilibrium model (Kapsenberg *et al.*, 2015). Target pH values for E1 were 8.2 (a low *p*CO_2_ treatment of ~264 μatm, representing the average pH of the current highest-pH month), 7.95 (a mid *p*CO_2_ treatment of ~500 μatm, representing both the average pH of the current lowest-pH month, as well as the average pH of the future highest-pH month) and 7.7 (a high *p*CO_2_ treatment of ~920 μatm, representing the average pH of the future lowest-pH month) (Table 1). Target pH values for E2 were equivalent to those of E1 for the mid and high *p*CO_2_ treatments; however, the low treatment was set at 8.11 pH units (~337 μatm) because the 8.2 pH value from E1 at 4°C resulted in an environmentally improbable *p*CO_2_ value (~265 μatm).

**Table 1:** Average seawater chemistry conditions during the 2-week experiments of E1 (−0.8°C) and E2 (4°C)

Treatment	*p*CO_2_ (μatm)	pH	Ω_arag_	Rationale for pH conditions
E1 (ambient temperature)				
low	315 ± 14	8.13 ± 0.02	1.71 ± 0.06	Present-Day Summer
mid	427 ± 11	8.00 ± 0.01	1.33 ± 0.02	Present-Day Winter/Future Summer
high	901 ± 34	7.71 ± 0.01	0.70 ± 0.02	Future Winter
E2 (elevated temperature)				
low	379 ± 16	8.07 ± 0.02	1.77 ± 0.06	Present-Day Summer
mid	513 ± 10	7.95 ± 0.01	1.39 ± 0.02	Future Summer
high	961 ± 14	7.69 ± 0.06	0.81 ± 0.01	Future Winter

Values are expressed at mean ± s.d. The rationale for pH conditions is based on environmental data and local modeling based on Kapsenberg *et al.* (2015).

**Figure 1:**
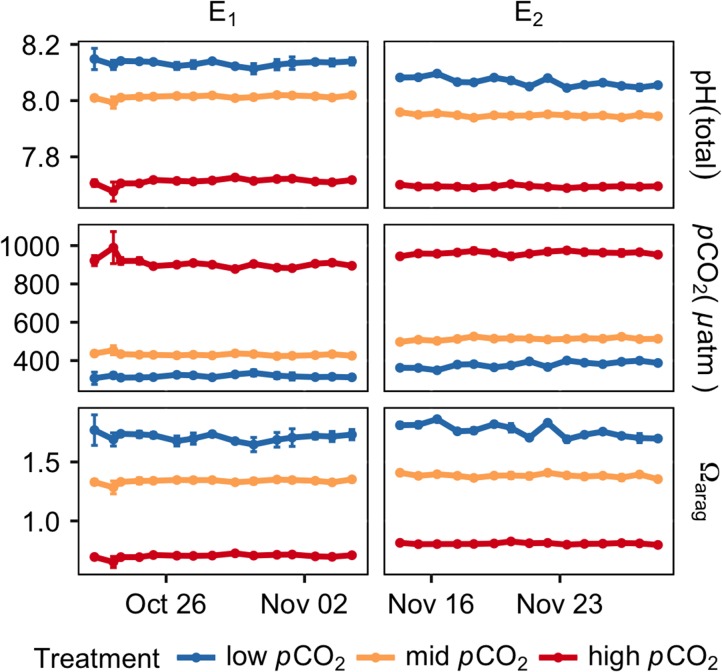
Seawater chemistry conditions throughout the 2-week experiments of E1 (left, −0.8°C) and E2 (right, 4°C). The average *p*CO_2_, as well as the Ω_arag_ values, are calculated from spectrophotometrically derived pH measured daily and total alkalinity collected every other day (data not shown). All error bars represent standard deviation over the three replicate tanks for each treatment.

The zooplankton culture system used for E1 and E2 consisted of 3 culture vessels per treatment (9 culture vessels total) submerged in seawater held at the experimental temperature (either −0.8°C or 4°C). Culture vessels were created by modifying clear 1-liter polycarbonate plastic tanks, accompanying lids and 400 μm mesh baffles that are often used in zebrafish research (Model AHLT3, Pentair Aquatic Ecosystems, Cary, NC, USA). To ensure that the animals were exposed to a continuous flow of *p*CO_2_ treated seawater, water input was regulated using irrigation button drippers (W221B, DIG Irrigation Products, Vista, CA, USA), at a flow rate of 2 liters per hour. The volume of one vessel was replaced on a 20-min basis. As a result, the *p*CO_2_ levels in the replicate culture vessels were highly similar within a treatment and tracked the reservoir *p*CO_2_ concentrations closely (see Results, Fig. 1). For each experiment, approximately 400 pteropods were randomly selected and added to each of the culture vessels. Only actively swimming individuals were taken for experiments. Individuals were not fed during these experiments.

### Carbonate chemistry analysis

Seawater chemistry was analyzed for the culturing tanks as previously described by Johnson *et al.* (2016). Water samples were fixed with 0.02% mercuric chloride and stored at 4°C until analyzed (Dickson *et al.*, 2007). Total alkalinity measurements were performed using an open-cell titration method (Mettler Toledo T50, Columbus, OH, USA) and pH readings were conducted using a *m*-Cresol spectrophotometric assay (Clayton and Byrne, 1993) conducted on a Shimadzu spectrometer (Model UV2501PC, Kyoto, Japan). Carbonate chemistry parameters were calculated using CO2calc (Robbins *et al.*, 2010) using constants from Lueker *et al.* (2000).

### Sampling

For E1 and E2, respirometry measurements were made at the start of each experiment (T_0_), and following mesocosm exposures of 24 h (T_1_), 48 h (T_2_), 4 days (T_4_), 7 days (T_7_) and 14 days (T_14_). At each timepoint, 18 individuals were randomly selected from culture vessels of each treatment and pooled for respirometry measurements. Individuals were not returned to the culturing tanks after respirometry was conducted, and thus each respirometry trial was conducted with different remaining individuals.

### Respirometry

Oxygen consumption rates were measured using a microplate system (Model SY1000, Loligo Systems, Viborg, Denmark). Each respirometry plate contained 24 closed-cell glass chambers with a 200 μL capacity, with a PreSens oxygen spot (PreSens, Regensburg, Germany) at the base of each chamber. Each plate was calibrated at two oxygen concentrations: 100% (aerated seawater) and 0% (1% Na_2_SO_3_ in milli-Q) at 2°C. All measured oxygen values were temperature-corrected to this calibration. At each timepoint, each plate was submerged in seawater (*p*CO_2_-treated water for E1 and E2, ambient seawater for E3), and a plastic transfer pipet was used to remove any visible air bubbles from the plate chambers. A single, visibly swimming pteropod was carefully added to a chamber using a transfer pipet, and the chamber was quickly sealed with a screw-on plastic cap lined with a PTFE seal. Care was taken to eliminate air bubbles from the plastic caps during the capping process. Six chambers per plate, spread across multiple rows and columns, were filled with only treatment seawater and were used as blanks to account for any background respiration due to microbial activity. In E1 and E2, the three plates were randomly assigned to a treatment in each timepoint to avoid any potential differences between plate calibrations (i.e. plate effects). Once every respirometry chamber was filled and sealed, each plate was then submerged in a flow-through acrylic water bath (Model CH10505, Loligo Systems) connected to a larger, temperature-controlled tank via a pump (Model MD7, Danner Manufacturing Inc, Islandia, NY, USA) that was held at the desired experiment temperature with a heat pump (IsoTemp 730, Fisher, Waltham, MA, USA). Although the temperatures were held as accurately as possible, the heat in the laboratory limited the lowest temperature of the respirometry system to −0.6°C for E1. Thus, for E1, respirometry was conducted 0.2°C higher than the experimental exposure temperature.

Each water bath was nested above an array of 24 optodes corresponding to the oxygen-sensitive spots in the respirometry chambers, which recorded optode phase values for each chamber every minute for approximately 6 h, or until oxygen concentrations fell below 60% saturation. Optode phase values were converted into oxygen concentrations using spreadsheets provided by PreSens. Following the oxygen measurements, pteropods were carefully removed and stored at −1°C until they were weighed. All individuals were visually inspected for mortality after the respirometry measurements were complete. Wet weight values were obtained by gently blotting individuals dry on a KimWipe (Sigma-Aldrich, St Louis, MO, USA) before transferring them to a microbalance (Cahn C-31; Thermo-Fisher, Waltham, MA, USA). Pteropods were then placed in a freeze dryer overnight (LabConco FreeZone, Kansas City, MO, USA) and reweighed to obtain dry weight values.

### Thermal tolerance

For E3, pteropods were kept at −0.8°C until the start of each respirometry run. Oxygen measurements were collected using the microplate respirometry system (described above) at several different temperatures: −0.8°C (approximate ambient temperature), 2°C, 4°C, 6°C, 8°C, 10°C, 12°C and 14°C. For each temperature, one plate was filled with pteropods (*n* = 18) and run for 6 h, or until oxygen concentrations fell below 60% saturation. Pteropods were then visually assessed for mortality and wet and dry weights were obtained (similar methods to E1 and E2).

### Data analysis

All statistical analyses were run in R (v.3.2.4). For each chamber, the rate of oxygen consumption over time was quantified by linear regression. For each microplate, the oxygen consumption rate of the six blank chambers were averaged and subtracted from the oxygen consumption rate of the sample chambers to account for any microbial respiration. There was a significantly different scaling coefficient between timepoints (ANCOVA, *P* = 0.003), but not between treatments (ANCOVA, *P* = 0.321), likely due to a change in dry weight over time. Thus, different scaling coefficients were derived for each timepoint in each experiment (E1 and E2), and used to correct pteropod respiration rates separately for each respective timepoint and treatment. Pteropod respiration rates were mass-corrected using the mean mass in each experiment (145.76 mg in E1, 142.21 µg in E2) using mass-correction equations from Steffensen *et al.* (1994). As the mass of organisms remained constant over E3, they were mass-corrected using a common scaling coefficient (α = 0.585), to a mean common mass (152.78 µg).

Oxygen consumption values were compared between treatments using two-way ANOVAs after testing for significance against a mixed-effect model (R package lmerTest v.2.0–32) which included individual glass chambers as a random effect to account for any sensor variation between each oxygen-sensitive spot. Post-hoc Tukey HSD tests were conducted to resolve pairwise differences between treatments and timepoints.

Q_10_ values were also calculated to compare metabolic rates (R_1_, R_2_) measured at different temperatures (T_1_, T_2_) as:

Q_10_ = (R_2_/R_1_)^10°/(T2-T1)^.

## Results

At a survivorship level, time in the culturing system tanks did not affect the juvenile pteropods during the experiment. We observed a very low percentage of mortality throughout the duration of E1. During E2 < 1% total mortality was observed during the first 8 days of the experiment, after which there was a ~4.4% daily mortality rate observed for all treatments until the termination of the experiment (Day 14). This E2 mortality rate was not significantly different between treatments (Cox proportional hazards regression model, *P* = 0.57).

Throughout the exposures of E1 and E2, carbonate chemistry remained highly stable (Fig. 1, Table 1). Seawater temperature also remained stable, with an average temperature of −0.97 ± 0.07°C during E1 and 3.97 ± 0.08°C during E2 (mean ± s.d.). Salinity was also maintained at stable levels: 35.9‰ for E1 and 35.5‰ for E2.

### Oxygen consumption (E1 and E2)

Oxygen depletion in the respirometry chambers occurred at very consistent rates, with samples being eliminated from the analysis *a priori* only if they exhibited an *r*^2^ < 0.8 when plotting oxygen consumption over time. Here, only 7 out of 611, or 1.1%, combined for E1 and E2 were eliminated from the analysis. As expected from the results of elevated temperature, average respiration rates for E2 were higher than those of E1 (Q_10_, averaging across all treatments and timepoints = 1.7; Q_10_ values will be further explored in E3 results).

**Figure 2:**
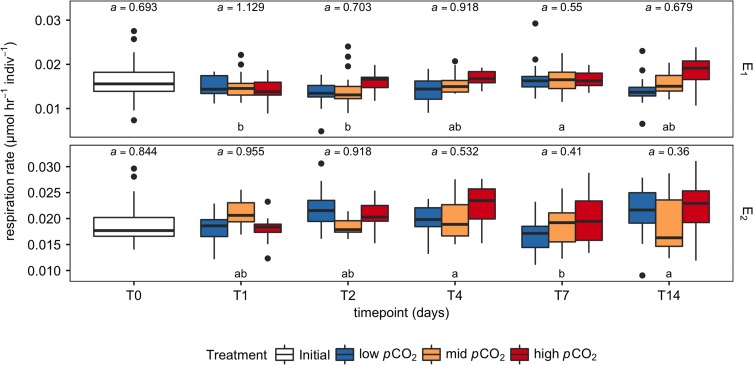
Oxygen consumption over time for each *p*CO_2_ treatment (low, mid, high), and temperature (E1, acclimated to −0.8°C and measured at −0.6°C; E2, 4°C). *a* denotes the scaling relationship used to mass-correct each respective timepoint, while letters indicate the Tukey post-hoc groupings between timepoints (*P* < 0.05), conducted separately for each experiment. Respiration rates are normalized to the mean mass for each experiment (45.76 µg in E1, 142.21 µg in E2).

Oxygen consumption was mass-corrected using different scaling coefficients for each timepoint and experiment (Fig. 2). These scaling coefficients decreased significantly over time in E2 (−0.04 day^−1^, *P* = 0.031), but did not show a significant directional trend over time in E1 (*P* = 0.417). All further respirometry results were conducted on mass-corrected values where these coefficients were used to correct individuals from each timepoint to that experiment’s mean body mass (145.76 mg for E1, 142.21 µg for E2). Analyses replicated using a common mean scaling coefficient for each experiment yielded similarly significant end results.

In E1, timepoint (F(4, 250) = 4.577, *P* = 0.001), treatment (F(2, 250) = 7.863, *P* < 0.001) and their interaction effect (F(8, 250) = 2.413, *P* = 0.016) were statistically significant. For the effect of treatment, a post-hoc Tukey test concluded that respiration rates in the high *p*CO_2_ treatment were significantly higher than the other two treatments. Respiration rates generally increased over time (Fig. 2). The interaction term likely refers to the emerging effect of treatment over time (Supplementary Fig. 1).

Similarly, in E2, timepoint (F(4, 251) = 4.636, *P* = 0.001), treatment (F(2, 251) = 4.782, *P* = 0.009) and their interaction effect (F(8, 251) = 4.059, *P* < 0.001) were significant. For the effect of treatment, a post-hoc Tukey test concluded that the respiration rates in the high *p*CO_2_ treatment were significantly higher than both that of the mid and low *p*CO_2_ treatment. The pattern of increasing respiration rates over time observed in E1 were not as apparent as in E2 (Fig. 2), and appeared to become more heterogeneous between timepoints over time. Similarly, the interaction term likely refers to the emerging effect of treatment over time (Supplementary Fig. 1).

### Oxygen consumption during acute high temperature exposures (E3)

**Figure 3:**
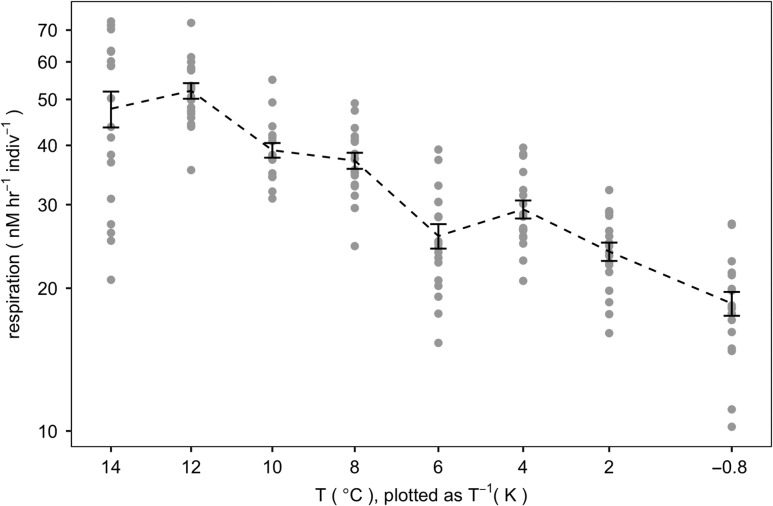
Respiration rate as a function of temperature (E3), plotted as an Arrehnius plot (inverse Kelvin temperature on the *x*-axis, log of respiration rate on the *y*-axis) with error bars indicating standard error. Respiration rate is expressed as nM h^−1^ indiv^-1^, and is mass-corrected to the average mass of the pteropods in E3.

Mortality was not observed during the 6-h acute temperature exposures and respirometry measurements conducted in E3. Across the observed temperature range (−0.8°C to 14°C), oxygen consumption increased predictably with temperature (Fig. 3) and was heterogeneous between tested temperatures (F(7, 135) = 35.47, *P* < 0.001). Although the mean respiration rate measured at 14°C was slightly lower than the mean respiration rate measured at 12°C, this difference was not significant (Tukey’s HSD, p_*adj*_ = 0.779), and thus there was no discernible critical temperature threshold. Similarly, a segmented linear regression conducted on an Arrhenius plot of the data (inverse of temperature (Kelvin) plotted against log_10_(respiration rate)) could not discern a breakpoint (R package ‘segmented’, v.0.5–1.4). The Q_10_ value calculated over the temperature extremes (−0.8 to 14°C) was 3.0 (Table 2).

**Table 2:** Calculated *Q*_10_ values determined for several ranges of temperatures

Temp	−0.8 to 2°C	−0.8°C to 4°C	−0.8°C to 6°C	−0.8°C to 8°C	−0.8°C to 10°C	−0.8°C to 12°C	−0.8°C to 14°C
Q_10_	2.5	3.5	2.0	3.3	3.1	3.9	3.0

### Weight

**Figure 4:**
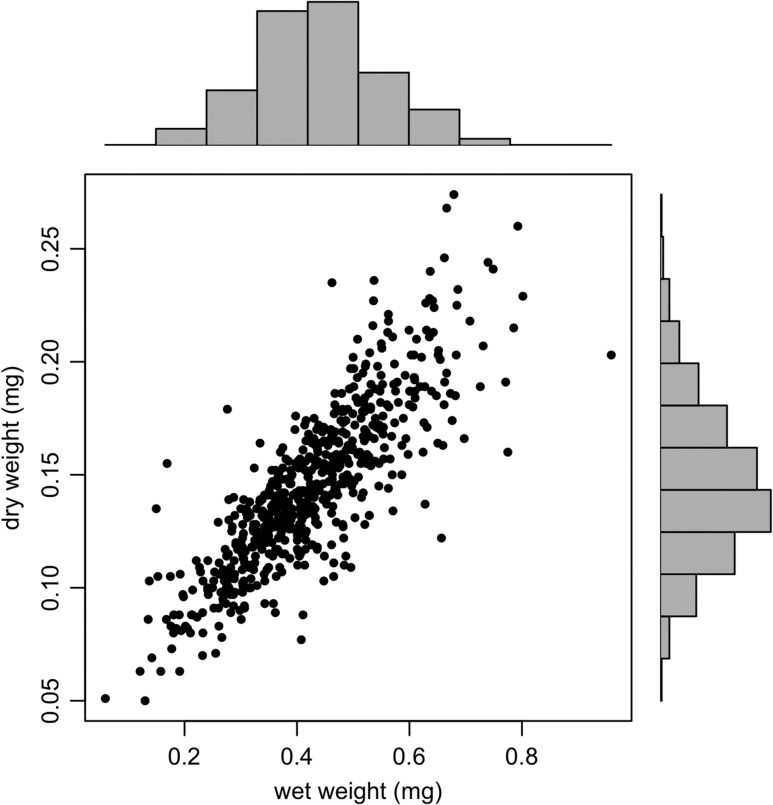
Correlation between pteropod wet weight and dry weight (each expressed in milligrams), with histograms along each axis. This correlation is linear (*r*^2^ = 0.69, *P* < 0.001).

**Figure 5:**
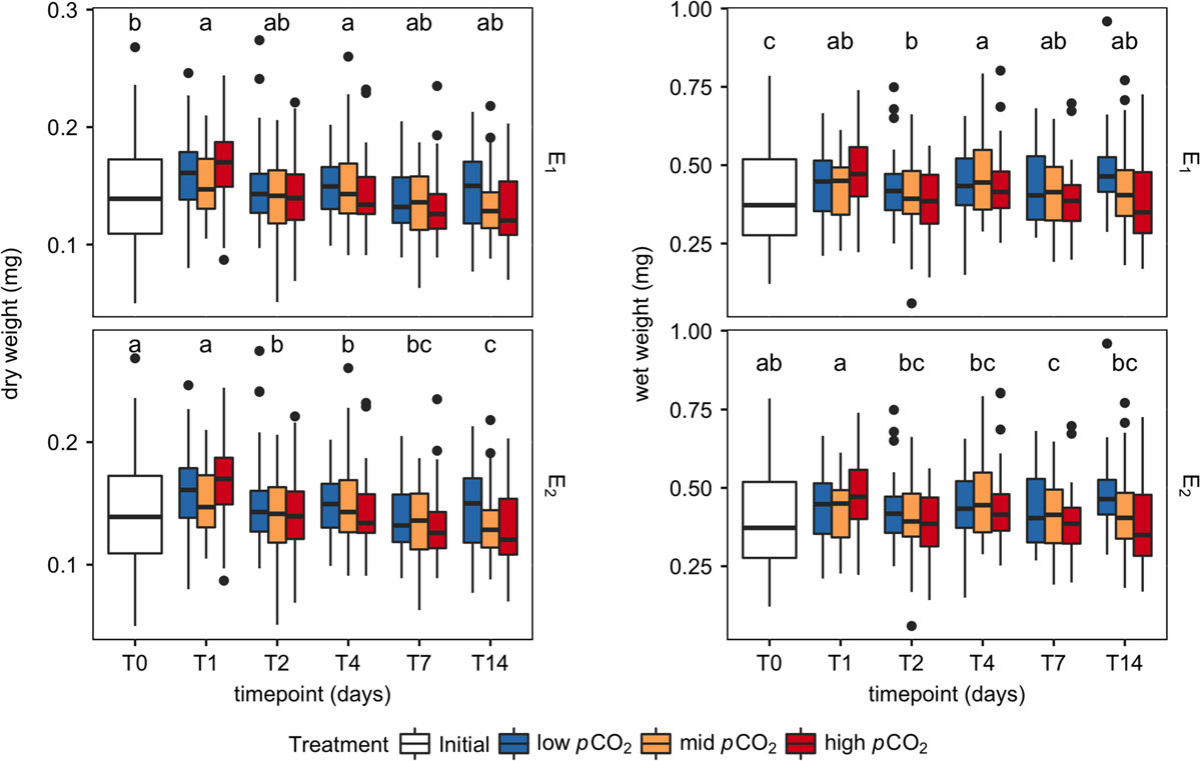
Pteropod dry and wet weights (mg) over time, for each *p*CO_2_ treatment (low, mid, high), and temperature (E1, −0.8°C; E2, 4°C). Letters represent post-hoc Tukey test groupings (*P* < 0.05) conducted separately for each weight type and experiment.

Generally, dry weights of pteropods agreed well with their respective wet weights for E1 and E2 (Fig. 4, *r*^2^ = 0.67). In E1, although there was no significant change in pteropod wet weight, dry weight of pteropods was heterogenous over time (Fig. 5) (F(5, 287) = 9.254, *P* < 0.001).

In E2, although there was a small significant effect of treatment on dry weight (F(2, 284) = 3.49, *P* = 0.03), there was a more significant effect of treatment on wet weight (F(2, 284) = 13.488, *P* < 0.001). For both dry and wet weight in E2, pteropods from the high *p*CO_2_ treatment had a lower average mass than the other treatments. At the same time, the interaction term between treatment and timepoint was only significant for wet weight (F(8, 284) = 2.69, *P* = 0.007), which fluctuated to a further degree during the experiment.

Mean dry and wet weights of pteropods in E3 were 0.152 mg and 0.452 mg, respectively, and there were no significant changes to either of these factors between temperature runs in E3 (ANOVAs, *P* = 0.39 and *P* = 0.46, respectively). The entirety of E3 used the same cohort of pteropods and all temperature exposures were completed within three days of collection from the field.

## Discussion

**Figure 6:**
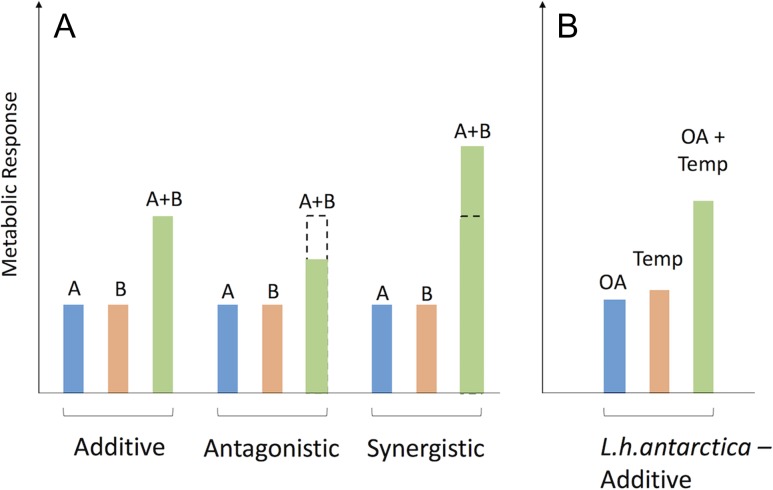
A conceptual diagram of multistressor interactions (**A**) in comparison with multistressor results for *L. h. antarctica*’s metabolic response to simulated OA and temperature stress (**B**). Dotted bars indicate the additive response. Antagonistic responses are lower than the additive response, while synergistic responses are higher than the additive response. Conceptual portion of the figure was adapted from Todgham and Stillman (2013).

Multiple stressors can have complex interacting effects on physiological processes in ectothermic animals (Gunderson *et al.*, 2016). Characterizing these organismal responses is critical to estimating the potential vulnerability of species to global change (Boyd, 2011; Todgham and Stillman, 2013; Gunderson *et al.*, 2016). In this study, we examined the metabolic rate of juvenile-stage pteropods in response to the interacting effects of *p*CO_2_ and temperature. We found that metabolic rate was increased by elevated *p*CO_2_ at both experimental temperatures. Our results show that the interaction of temperature and *p*CO_2_ elicited an additive response in juvenile pteropods (Fig. 6).

Specifically, at ambient temperature (−0.8°C, E1), pteropods exposed to the high *p*CO_2_ treatment exhibited elevated respiration rates relative to those exposed to low *p*CO_2_ conditions. This increase in metabolic rate is consistent with a bioenergetic outcome with increased costs of maintenance (Sokolova, 2013), possibly due to *L. h. antarctica’s* ability to self-repair its shell from the inside by secreting aragonite in response to physical scarring caused by undersaturation (Peck *et al.*, 2016). As the shell’s function is to protect the pteropods, as well as to help regulate their buoyancy, its repair is a critical component of their energy budget under future undersaturated conditions of OA, and evidence of dissolution on shells has been documented on Antarctic pteropods collected from the wild (Johnson *et al.*, 2016). The increase in metabolic rate as a response to high *p*CO_2_ may also be due to increased energetic demand for acid-base balance (Sokolova, 2013), which may be critical in providing an internal environment for this critical aragonite shell repair.

An effect of *p*CO_2_ exposure on metabolic rate was also observed in E2, where pteropods exposed to the high *p*CO_2_ treatment exhibited elevated respiration rates relative to those exposed to low and mid *p*CO_2_ conditions. The effect of temperature alone resulted in 1.29-fold higher respiration rates in E2 than E1 (over a 4.6°C temperature change, or Q_10_ = 1.752). Although E1 and E2 cannot be directly compared because the animals used were from different collection days, incorporating respirometry results into a model shows that there is no significant interaction term between *p*CO_2_ treatment and temperature (*P* = 0.309). The effect of elevated CO_2_ did not change drastically between temperature treatments (high *p*CO_2_ treated organisms had 1.112-fold higher respiration rates in E1, and 1.081-fold higher respiration rates in E2 when compared to low *p*CO_2_-treated organisms). These trends designate the observed interaction as additive—where the sum effect of two stressors is equal to the sum of the effects of the individual stressors. In contrast, antagonistic effects yield an effect less than the sum of the individual stressors, while synergistic effects yield an effect greater than the sum of the individual stressors (Fig. 6). In this case, neither *p*CO_2_ or temperature are the key driver—they act on similar scales and do not exhibit an interaction term.

We also observed that the variance in metabolic rate increased over time in every treatment in E2 (Fig. 2b). This does introduce some moderate heteroskedasticity into the analysis (Breush Pagan test, R package lmTest v.0.9–35, *P* < 0.0001) which cannot fully be corrected by a Box-Cox transformation and yielded similarly significant results when run through the same model. Thus, we present the data as uncorrected. This increase in variation could be due to a variation in response to high *p*CO_2_ emerging through time, or to different degrees of starvation over the experimental period that interacted with the *p*CO_2_ response and manifested itself as an increase in variance.

Due to the large range of body sizes in E1 and E2, these analyses were conducted after accounting for the contribution of mass to metabolic rate. This was done by decreasing per-individual respiration rates of larger pteropods, and increasing per-individual respiration rates of smaller pteropods following a calculated scaling coefficient to eliminate the mass-dependency of respiration rate (Steffensen *et al.*, 1994). However, pteropod mass decreased throughout the experiments, and especially began to deviate between treatments in E2 (Fig. 5). The general weight decrease is most likely due to the starvation of the pteropods during our two-week long incubations, as the tanks in our culturing system were fed from a filtered seawater intake. It was not logistically feasible to account for a feeding regimen in our limited season length in the Antarctic. The difference in mass between treatments by the end of the experiment mirrors our expectations based on our measurement of respiration rate; that is, the individuals for the high *p*CO_2_ treatment had a decreased body mass over time, possibly due to their increased metabolic demand in a low-pH environment causing them to burn through energy reserves. Although dry weights of sampled pteropods were significantly different between treatments by the end of E2, as there was no difference in the scaling coefficient (ANCOVA, *P* = 0.321) between treatments, scaling coefficients were calculated discretely for each timepoint of each experiment, then applied to correct the respiration rate to an average body mass for each experiment.

Logistically, it was necessary to conduct sequential plankton tows to collect animals for the respirometry trials. Thus, it is important to note that pteropods from E1 and E2 were collected 23 days apart and represent different cohorts of field-collected individuals. Although water chemistry at the collection site remained fairly stable during this time (Johnson *et al.*, 2016) and initial body mass was similar between collections, austral spring bridges winter dormancy and summer time growth, and pteropods could undergo drastic transcriptomic changes during this season (Johnson, 2017). Overall, conducting E1 and E2 concurrently with the same cohort of collected individuals was not logistically feasible due to field collection and laboratory tank space constraints in a remote field location.

In general, studies that have examined the temperature/*p*CO_2_ multistressor scenario in marine metazoans have found that antagonistic effects, along with additive effects, are far more common than synergistic effects in adult marine invertebrates (Byrne and Przeslawski, 2013) and are also relatively common in early life history stages (Przeslawski *et al.*, 2015). Indeed, it is becoming increasingly clear that there is no unifying theory regarding multistressor effects on a physiological level, and that species-to-species variation can affect these interactions significantly (Lefevre, 2016).

In this additive interaction, we found that temperature and *p*CO_2_ stress operated on a similar scale—a result which contrasts research on other Antarctic ectothermic species. In studies on early stage Antarctic dragonfish, *Gymnodraco acuticeps*, increased temperature significantly increased development rates and embryonic metabolic rate (Flynn *et al.*, 2015). However, although *p*CO_2_ stress alone did not negatively affect embryo physiology, there was a synergistic interaction when embryos were exposed to both high *p*CO_2_ and high temperature. Similarly, a study rearing larvae of the sea urchin, *Sterechinus neumayeri*, in *p*CO_2_ and temperature treatments found that there was no effect of either individual stressor on the ability to withstand acute larval heat stress (Kapsenberg and Hofmann, 2014). However, rearing urchin larvae at both high *p*CO_2_ and high temperature resulted in a decreased ability to tolerate acute heat stress at certain early developmental stages. Overall, studies seem to indicate that multistressor relationships in the Antarctic can vary between species, and that this interaction can occasionally make certain species less vulnerable to experimental conditions of ocean change than previous studies have suggested (Peck, 2005).

Although metabolic rates of polar pteropod species are highly understudied, there have been three other studies that have observed the effects of lab-manipulated *p*CO_2_ on *Limacina spp*., only one of which was conducted in the Antarctic. In the northern hemisphere, studies collecting pteropods from Kongsfjord, Spitsbergen have found varying effects of *p*CO_2_ and temperature based on collection time, species and acclimation duration. Specifically, Lischka and colleagues generally found an increase in MO_2_ with increasing temperature and *p*CO_2_ in acute exposures of *L. h. helicina* collected in January and February, with the exception of the highest-temperature (6.8°C), where an intermediate *p*CO_2_ (650, out of a range of 180–880) caused the highest increase in metabolism (Lischka and Riebesell, 2017). This ‘hormesis-type’ effect, where an intermediate treatment yielded the highest metabolic increase, was prominent at all temperatures during the paired 9-day acclimation experiment. In contrast, *L. h. helicina* collected from the same site in May–June showed no metabolic response to *p*CO_2_ manipulation at ambient temperature (0°C), but there was an observed increase in respiration rate when increasing *p*CO_2_ at high temperature (4°C) after a 24-h incubation (Comeau *et al.*, 2010). The hormesis-type effect described in Lischka and Riebesell (2017) was not observed in our study in either experiment. However, Lischka and Riebesell (2017) also observed different multistressor effects in the congener *L. retroversa* collected at the same site, which did not exhibit any interaction between temperature and *p*CO_2_ effects. Altogether, these studies in the Arctic show marked differences in pteropod response to these multistressor conditions at different acclimation durations and collection times—even in closely related species. Specifically, the seasonal differences are likely a situation that, while not unique to polar ecosystems, may be strengthened there due to the strong seasonality experienced at high latitudes compounded by the collection of different life history stages at different times of the year.

In contrast, Seibel and colleagues resolved a metabolic suppression under high CO_2_ at concentrations analogous to the ‘low’ and ‘high’ treatments utilized in E1 (Seibel *et al.*, 2012). While Seibel and colleagues collected *L. h. antarctica* near our collection site (the three sites are 25 km, 35 km and 75 km north), they were collected far later in the austral summer. This is reflected in the pteropod mass—the pteropods collected for E1 and E2 in October and November 2015 (0.05–0.95 mg) for our study were less than 10% of the mass of those collected by Seibel *et al.* in January and February (0.8–15 mg). Although seasonal development of pteropods in the Antarctic is poorly documented, this growth spurt coincides with phytoplankton blooms in the Ross Sea region (Goffart *et al.*, 1999). This corroborates previous studies that suggest *L. h. antarctica* potentially exhibit a 1-year life cycle, spawning and senescing in late summer (Hunt *et al.*, 2008). The fact that this phytoplankton bloom plays a large part in pteropod phenology, growth and development is supported by Seibel *et al.*’s disparate results between replicate experiments conducted in consecutive years, which is hypothesized to be a function of low ocean chlorophyll abundance already suppressing metabolism in the second year of trials. Given that phenology and interactions with seasonality have been seen as key factors in governing the Arctic pteropod’s response to *p*CO_2_ and temperature, we posit that these factors drove the differences between the results in this study and that of Seibel and colleagues.

In the case of seasonality, observations of pH in McMurdo Sound show austral summer (January and February) as the least acidic period, with a strong seasonal shift towards most acidic conditions in the austral winter (Kapsenberg *et al.*, 2015). This is most likely caused by the stark seasonal day/night cycles at high latitudes, which result in 24-h darkness during the winter and 24-h daylight during the summer. Considering the high pH seasonality in the Ross Sea region, it is probable that pteropods at different life stages during a different time of year will exhibit a disparate response to *p*CO_2_ stress. Furthermore, different life stages will experience different magnitudes and duration of *p*CO_2_ stress, both of which have been seen as critical factors in determining calcification capacity in pteropods collected from the wild in the U.S. Pacific Northwest (Bednaršek *et al.*, 2017). This highlights the importance of considering life stage, phenology and current-day seasonal variation of abiotic stressors when studying global change biology, as impacts during juvenile stages may also be difficult to recover from (Hettinger *et al.*, 2012, Manno *et al.*, 2017). If hypercapnia elicits a different response at different life stages, the seasonal trend of pH in McMurdo Sound may play an integral part in this organism’s future viability under environmental change.

To contextualize our high-temperature experiment (E2) in the scope of *L. h. antarctica*’s thermal tolerance, we measured respiration rates at ambient *p*CO_2_ levels at increased temperatures (E3) to find that individuals tolerate temperatures far higher than their current, natural environment. The McMurdo Sound region does not see surface temperatures above 0°C (Cziko *et al.*, 2014), yet respiration rates of *L. h. antarctica* increased predictably with temperature following the Arrhenius equation (Alcaraz *et al.*, 2013) up through 14°C. However, it is the case that these acute temperature exposures do not account for every aspect of animal physiology, such as acclimation, and it is possible that growth, energetics and reproduction cannot keep pace at these higher temperatures. Although the experimental temperature exposures of 4° in E2 is well within the acute thermal tolerance of *L. helicina antarctica*, it is possible that, especially in conjunction with the additive stressor of OA, pteropod populations in the Southern Ocean may be negatively impacted in the near future.

Changes in resting metabolic rate can play a critical role in an organism’s energy budget, potentially drawing resources away from other energetically costly processes, such as growth and reproduction. In this light, any changes in respiration rate in response to multiple environmental stressors may be critical for the fate of marine organisms in the near future. Additive responses, such as those seen here (Fig. 6), reflect the results of individual stressor experiments in a multistressor scenario. Other species may exhibit a more or less extreme response. While it is clearly unfeasible, in many regions or taxa, to design experiments that will fully simulate all parameters of future oceans, focusing on a multitude of potentially harmful or co-occurring stressors may be necessary to fully tease apart the fate of future oceans.

Globally, marine ecosystems are projected to face multiple, concurrently changing variables in the future, with abiotic factors that are unique to a particular habitat type and region, of which OA is just one stressor (Breitburg *et al.*, 2015). Emergent examples of this are scenarios of OA and thermal stress in coral reef ecosystems (Hoegh-Guldberg *et al.*, 2007) or persistently covarying stressors like OA and hypoxia in temperate kelp forest ecosystems (Frieder *et al.*, 2012). Further study of the energetics of marine organisms in response to potentially interacting abiotic factors may lend additional insight into understanding the vulnerability of species to future ocean change. Eventually, investigating the root cause and patterns that lead to certain species exhibiting additive effects may not only allow for a better understanding of stress physiology, but may also allow for a critical, more accurate glimpse of future community dynamics in a changing world.

## Supplementary Material

Supplementary Data

## Data Availability

The data package for this study is available on Dryad at the following: doi:10.5061/dryad.dh078.
